# Customized Allogeneic Bone Augmentation Improves Esthetic Outcome in Anteromaxillary Dental Implantation

**DOI:** 10.1155/2022/6943930

**Published:** 2022-03-22

**Authors:** Manfred Nilius, Minou Hélène Nilius, Charlotte Mueller, Bernhard Weiland, Dominik Haim, Anna Krahe, Guenter Lauer

**Affiliations:** ^1^Niliusklinik, Londoner Bogen 6, D-44269 Dortmund, Germany; ^2^Department of Oral and Maxillofacial Surgery, University Hospital “Carl Gustav Carus”, Technische Universität Dresden, Fetscherstr 74, D-01307 Dresden, Germany

## Abstract

**Purpose:**

In cases of severe atrophic maxilla or maxillary involution, augmentation is necessary for implant-supported prosthetics. Using bone grafts is a standard procedure, and using customized allogeneic bone blocks may be a predictable alternative before dental implantation. *Clinical Findings*. This case study shows the digital workflow, including a preimplantological augmentation by a customized allogeneic block, followed by soft tissue optimization and template-based dental implantation, after six months of healing. It is part of a three-year follow-up study on the resorption rate of allogeneic bone blocks. *Outcomes*. Allogeneic bone augmentation is an alternative treatment option to autologous bone grafts. It allows predictable advanced backward planning (ABP) even in the maxillary esthetic zone. Diameter-reduced implants show long-term stability of a minimum of three years after loading and excellent results of prosthetic fixtures.

**Conclusion:**

Prefabricated customized allogeneic blocks for augmentation may increase the fitting accuracy of the graft, decrease morbidity, and reduce the operation time in esthetic maxillary rehabilitation.

## 1. Introduction

In the case of maxillary involution, augmentation is necessary for implant-supported prosthetics. Using bone grafts is a standard procedure, and using customized allogeneic bone blocks may be a predictable alternative before dental implantation.

An augmentative procedure for the hard or soft tissue is necessary and represents a critical success factor in implantology in around half of all implantological interventions. To decide which bone substitute material is the most suitable depends on the indication and the individual wishes of the patient and surgeon. Besides autologous bone, the focus is increasingly on allogeneic transplants, which have been used since 2007 [1].

Reduced morbidity and pain potential associated with the transplant removal are particular advantages of using an allogeneic transplant; thus, the process is more pleasant for the patient. Furthermore, we can avoid many complications such as wound healing disorders and uncontrolled resorption using an allogeneic transplant, and allogeneic materials, in contrast to autologous materials, are virtually unlimited [2]. They are similar to autologous bones in terms of requirements and functional profile, and the prerequisites for bone regeneration are met [3–6]. The overall rehabilitation in “backward planning” is based on the bony situation. Thus, the ideal positioning of the subsequent implants is known before augmentation.

## 2. Case Presentation

A 65-year-old female patient presented for an implantological consultation to replace the existing bridge and replace a missing lateral incisor (tooth 12), which had been in situ for 19 years, with a single-tooth restoration. Tooth 12 was lost due to cystectomy three decades earlier with subsequent bone regression.

## 3. Clinical Findings

**Figure 1:**
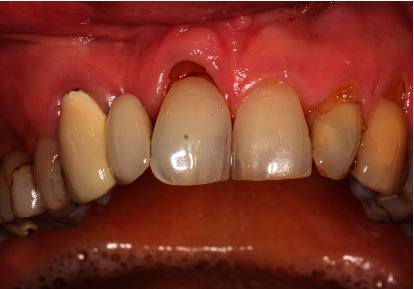
Initial situation with mesial trailer bridge on tooth 13 with missing tooth 12.

**Figure 2:**
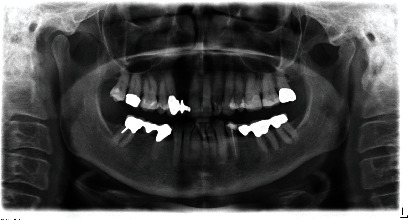
The orthopantomogram (OPT) shows a bone defect in the region of the right lateral incisor.

Clinically, there was a mesial trailer bridge on tooth 13 with mesial support on tooth 11. A vestibular soft tissue recession and erosion at the enamel-cement junction were evident on this tooth. In region 12, a vertical and transverse bone deficit was already clinically evident, which was confirmed by the radiological diagnosis (Figures 1 and 2).

The orthopantomogram showed a bone defect in region 12 and moderate horizontal, molar bone loss without vertical fractures. Teeth 35 and 46 are missing, and teeth 34 and 47 had endodontic and prosthetic treatment. Cone-beam computed tomography (CB-CT) showed that the transverse width of the bone in region 12 was 4.5 mm.

## 4. Diagnostic Assessment

The patient wanted a single-tooth restoration. We recommended advanced backward planning (ABP), including preprosthetic bone and soft tissue augmentation. The alternatives would have been to renew with different material combinations, use removable dentures, or leave the extraction site without prosthetics (“courage to gap”).

**Figure 3:**
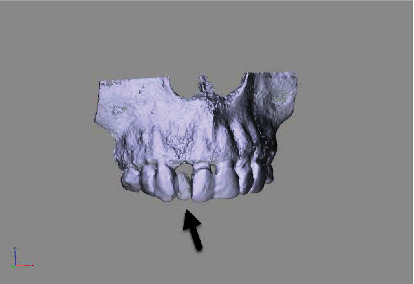
Rendered 3D planning model (STL file) showing a bony deficit in region 12.

**Figure 4:**
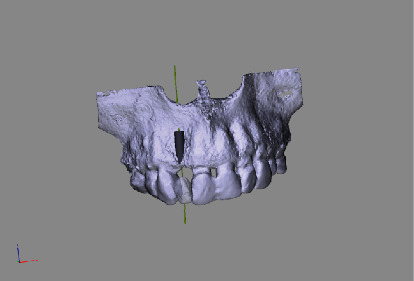
Rendered 3D planning model (STL file) showing a virtual implant in position 12.

**Figure 5:**
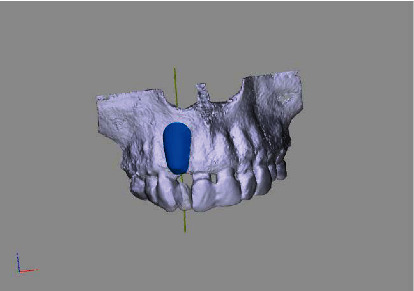
Rendered 3D planning model (STL file) with 3D bonebuilder block graft (3D BB) in position 12.

**Figure 6:**
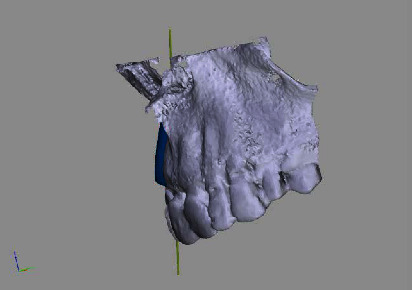
Rendered 3D planning model (STL file) with 3D BB in the transversal view.

**Figure 7:**
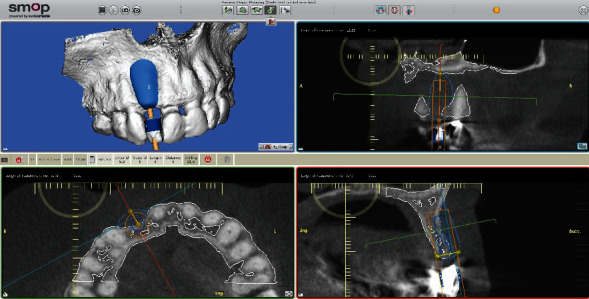
A planning tool for implant positioning and producing a SMOP template (Smop, Swissmeda, Baar, Switzerland) for navigated implant placement. Multiplanar view with 3D BB superimposed on the implant position.

**Figure 8:**
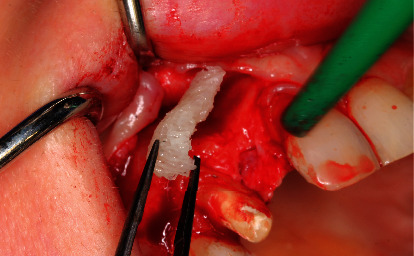
Intraoral view after opening the mucoperiosteal flap and inserting the individually CAD/CAM-milled block.

The concept of “ABP” means that one starts with the desired result and plans the work steps “backward” in a team with the dentist, implantologist, and dental laboratory. We decided for a treatment with a CAD/CAM manufactured allogenic bone block (maxgraft® bonebuilder, botiss biomaterials GmbH) and transferred CB-CT data to botiss biomaterials GmbH to virtually design the shape of a customized bone block to be able to maintain correct implant prosthetic planning, followed by ABP through digital wax-up of the future tooth crown 12. Next, we planned for Straumann implants (BLT Roxolid Ø 3.3 mm and length 12 mm; Narrow CrossFit platform). The ideal implant position and needed block volume was decided by determination of tooth axis and natural bone width for the upcoming implantation using 3D planning tools (Figures 3–8).

Based on CB-CT, botiss used 3D planning software to construct a model of the jaw defect and created the 3D bone block (maxgraft bonebuilder®, botiss biomaterials GmbH). The virtually created bone graft has to be checked by the doctor before the milling process, using a 3D PDF document, and modified if necessary. Once released, the production of the customized block graft begins. Each customized block graft is milled out of a processed freeze-dried cancellous bone block, originating from femoral heads from living donors obtained during arthroplastic surgery (Allotec process, Cells + Tissuebank Austria, Krems an der Donau, Austria). The delivery time is about four weeks.

## 5. Therapeutic Interventions/Timeline

### 5.1. Hard Tissue Augmentation (0–6 Months)

After removing the bridge on tooth 13, we augmented the bone by approaching through the marginal incision with mesial relief to expose the bone defect. The customized block graft was previously soaked in metronidazole to ventilate. Punctiform drilling to provoke bleeding of the local cancellous bone is necessary after trying this on. We fixed the bone block using two osteosynthesis screws (1.5 × 10 mm). A resorbable porcine pericardium membrane (Jason® membrane, 15 × 20 mm) was used to protect the augmentation (Figure 8).

### 5.2. Soft Tissue Augmentation (4th Month)

**Figure 9:**
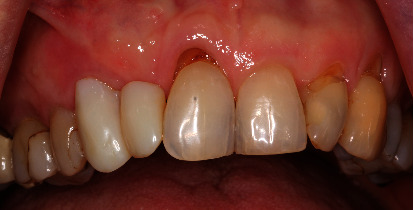
Intraoral view with long-term temporary prosthetics (PMMA) bridge 13-12 in situ.

**Figure 10:**
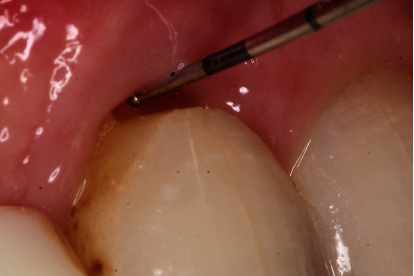
Intraoral view shows cervical recession of 2 mm on tooth 11.

Soft tissue optimization is necessary after around four months from bony augmentation; thus, we recommended connective tissue transplantation due to the recession on tooth 11 and the deficit of the attached gingiva in region 12. Donor site incision was made palatally of teeth 23 to 26 (Figures 9 and 10).

We tunneled the recipient gingiva region 11 to 12 without a vertical incision. Next, to accommodate the soft tissue graft, we prepared a supraperiosteal pocket. We stabilized a connective tissue graft transmucosal through a suture (5/0 PTFE, nonabsorbable suture material made of polytetrafluoroethylene, Teflon). Finally, we covered the recession on tooth 11 by the graft and a coronal transposition flap using a double-crossed suture.

On the first day after the operation, the recessions were slightly excessively covered by the graft. However, the soft tissue conditions were stable at the checkup after one month so that stable transplant healing could be assumed.

### 5.3. Dental Implantation (9th Month)

Six months after bone grafting and two months after soft tissue augmentation, dental implantation was performed. After opening the mucous membrane, we removed the osteosynthesis screws. The cancellous bleeding indicated that the bone had healed well.

**Figure 11:**
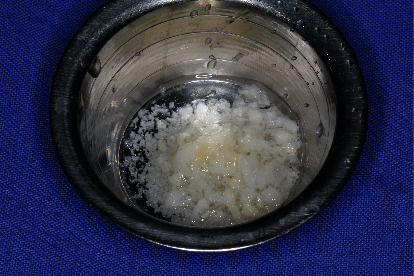
Processed human allograft (Maxgraft®; corticocancellous granules; Cells + Tissuebank Austria, Krems a. d. Donau, Austria).

**Figure 12:**
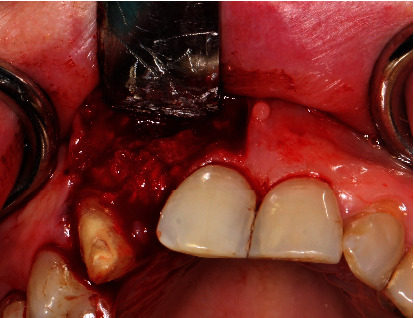
Superimposed processed human allograft covering the dental implant.

**Figure 13:**
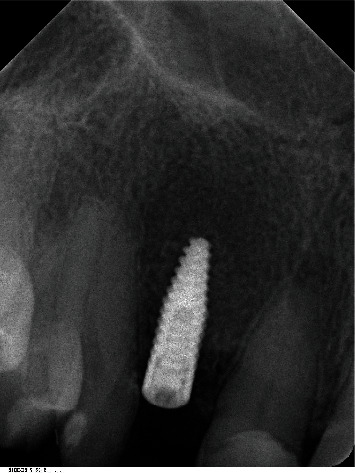
Dental X-ray after dental implantation.

As planned, we drilled a reduced-diameter bone level tapered implant (Straumann) made of Roxolid Ø 3.3 mm and length 12 mm, with a Narrow CrossFit platform with a maximum torque of 35 Ncm. Finally, we covered the implant region with particulate allogeneic bone substitute material (maxgraft® processed human allograft, botiss biomaterials GmbH) followed by saliva-tight wound closure. After intervention, we checked the dental implant's position using X-ray (Figures 11–13).

### 5.4. Implant Prosthetic Restoration (10th Month)

**Figure 14:**
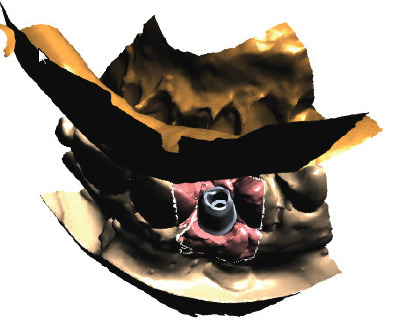
Design of the abutment/crown construction using 3Shape Dental Manager. Plaster model with the stationary scanner D2000 (3Shape, Trios). Antagonistic view.

**Figure 15:**
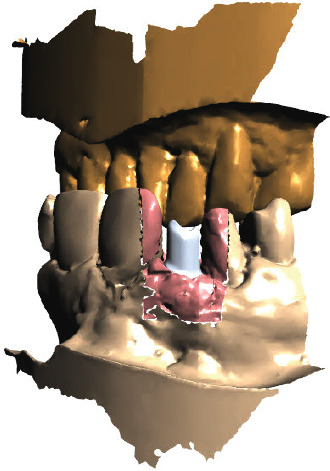
Design of the abutment/crown construction using 3Shape Dental Manager. Plaster model with the stationary scanner D2000 (3Shape, Trios). Lateral view.

We made a transgingival impression of the osseointegrated implant with an impression tray using the open impression technique three months after implantation. Then, to accommodate the future crown, we attached an individual abutment made of zirconium dioxide (Straumann CARES CAD/CAM) to the implant (Figures 14 and 15).

**Figure 16:**
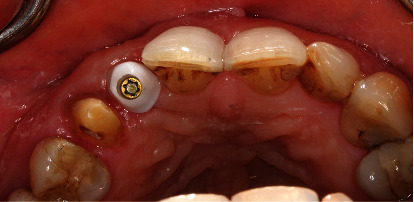
Custom-made implant abutment made of zirconium dioxide (Straumann CARES CAD/CAM).

**Figure 17:**
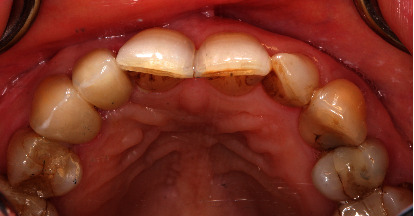
Implant prosthetic result after augmentation with 3D BB, implantation of a BLT implant with CARES abutment, and ceramic crown (top supervision).

**Figure 18:**
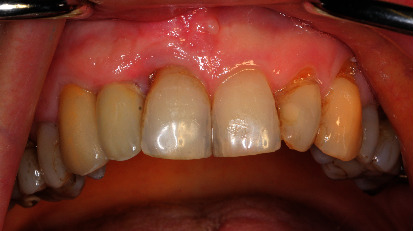
Implant prosthetic result after augmentation with 3D BB, implantation of a BLT implant with CARES abutment, and ceramic crown (anterior view).

**Figure 19:**
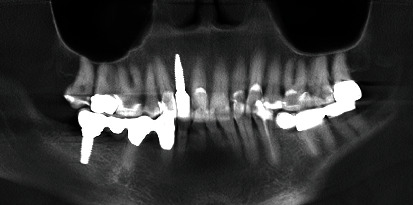
CB-CT after completion of the surgical and implant prosthetic rehabilitation.

We checked the chewing function, phonetics, and aesthetic try-on with correcting the tooth shape, tooth color, and transparency. After completion, we incorporated the tooth crown using dual-curing cement (Figures 16–18) and made a final CB-CT (Figure 19.

## 6. Discussion

The efficacy of processed allografts is comparable to autogenic/autologous bone transplants [7, 8]. However, processed allografts belong to bone substitutes, so comparing other xenogeneic or synthetical origin substitutes is reasonable [9]. There are a few animal studies of allografts because human-processed bone substitutes in animals are classified as xenogeneic transplant (usually immunological reactions caused by remaining collagen) [10]. Schmitt et al. [11] compared different bone substitutes and reported that there is no significant difference in bone formation rates between allogeneic material (35.4 ± 2.8%) and autogenous bone (42.7% ± 2.1%) in the maxillary sinus lift.

Moreover, the advantage of mineralized allografts compared to the deproteinized xenogeneic bone matrix was reported by Froum et al. [12]. Both variants are superior to a bovine demineralized bone matrix (24.9 ± 5.67%). Furthermore, autogenous bone also showed higher bone formation rates compared with a biphasic synthetic bone substitute (30.3 ± 2.2%).

26–32 weeks after sinus floor elevation, there were apparent differences in bone formation rates between allografts (28.3%) and xenografts (12.4%) [12]. Even the remaining nonvitalized bone substitute was better in allografts (33%) than that in xenografts (7.7%). Laino et al. [13] concluded that allogeneic blocks should be preferable because of the absence of extraction morbidity at the bone graft extraction site and less invasiveness. Their survey for sandwich osteotomy of the lateral lower jaw showed no notable differences between allogeneic blocks (30.6 ± 3.7%) and autogenous chin blocks (31.47 ± 2.2%). The results are consistent with the results from Laino et al. (2014). When comparing allogeneic sandwich osteotomies to onlay augmentation, the latter showed a higher complication rate due to increased soft tissue dehiscences.

When the healing period is uneventful and lasts longer (seven months), one can obtain a two-millimeter higher vertical augmentation using onlay plasty. Other authors confirmed these positive results [14–17].

Tallarico et al. [18] presented a fully digitized approach for the aesthetic zone using a customized 3D-printed titanium mesh. In analogy to 3D BB technology, they designed the scaffold according to the maxillary bone deficit by mirroring the contralateral and unaffected surface via marketed membrane by CB-CT. They used a 50 : 50 mixture of autogenous bone and bone substitute (Bio-Oss, Geistlich Biomaterials Italia, Thiene, Vicenza, Italy). An advantage of this method is simultaneous implant placement during augmentation. Additionally, a narrow-diameter implant helps avoid bone defects on the buccal side. After one year, the prosthetics became stable [18].

Nevertheless, removing the titanium mesh and the screws increases the surgical effort and makes the reapproach worse. A recently published case series with a 3-year follow-up period compared different methods of augmentation before dental implantation, including autologous bone graft (hip graft), 3D BB, and cortical plate [19]. They concluded that the use of allogeneic blocks for maxillary augmentation was comparable with autologous bone grafts. Bone height was stable for a minimum of three years after loading with resorption less than 10% in vertical, buccolingual, and mesiodistal directions. Short or narrow implants allow for the long-term stability of prosthetic fixtures. Furthermore, filling up the scaffold maintenance of the mesh could also be a helpful method since there is no donor site comorbidity.

The risk assessment of mineralized processed allografts follows high standards. Producers must comply with providing security for the patient, including a protocol of the treatment process and the original harvested material. Industrial processing of the material is aimed at eliminating allergenic and infective parts. Different chemical techniques are used by tissue banks, such as peracetic acid-ethanol treatment, thermal disinfection (elimination of potentially contagious agents), lyophilization, osmotic treatment with saline solution, treatment with acetone, and oxygen (elimination of cellular components and fats), and gamma-sterilization to guarantee safe and sterile allografts. Risks (i.e., the transmission of infection and antigenicity) reduce significantly due to the processing and adverse reactions and events related to allografts are monitored worldwide [20, 21]. Fretwurst et al. reported in an in vitro study that there was no similar immunological reaction using allografts but they found isolated cell residues and DNA parts within the matrix structure of different mineralized, decellularized allografts in block shape [22]. However, it lacks the clinical implications of these findings. Apart from considering risks associated with the origin and processing of the tissue, one must also consider surgical as well as patient-related risks based on the surgeons' experience or patients' compliance. Tension-free closure of the wound by “loop” or “pulley” vertical mattress suture is one of the criteria for the success of the treatment protocol and can lower the risk of complication because insufficient soft tissue can lead to opening of the incision line over the grafted bone [2]. We recommend ablating exposed allogenic areas spaciously.

The trabecular structure of the cancellous allogenic bone allows comparatively fast revascularization [23]. In this case here, reentry was performed after six months and the well-established bone was found, similar to other reports about allogenic bone blocks [6]. Today, nonabsorbable membranes made of 100% dense polytetrafluoroethylene (PTFE) are available. Furthermore, we recommend using titan-reinforced PTFE membranes, which have advantages for further implant-supported prosthetic planning. These membranes are an efficient barrier against cellular penetration and reduce wound dehiscence risk because of their small pore size. Long-time exposition of the membrane is possible. It is not comparable to fully developed keratinized soft tissue, but it protects the bone block. In this case, however, we opted for Jason membrane because at the same time a connective tissue graft was used for the neighboring tooth.

### 6.1. Dental Implantation

A drill template can be made based on the block planning data (3D BB). Bone resorption of allogenic bone blocks is reported to reach up to 5-10% after six months of healing; however, it can vary individually [24]. Six months after insertion, digital implant planning was done. The visualization of the bone was not ideal because of the incomplete vascularization. Allogeneic transplants' visibility is better after coverage by a radiopaque titan mesh membrane or a thin layer of radiopaque bone substitute in advance. Alternatively, by setting the Hounsfield units at the 200–400 HU range, differentiation of the reconstructed bone is relatively visible [19].

Usually, there is an attempt to insert the implants while using the local bone underneath the augmented area. Furthermore, there is a high risk of lifting the bone block during implant insertion because of the loose joint between the transplant and the local bone. Moreover, resorption, which is about 10 to 15%, is comparable with native bone grafts [1].

## 7. Conclusion

Allogeneic transplants are a suitable alternative to autologous bone. They enable a less invasive procedure compared to augmentations with the autologous bone, do not burden the patient with the removal of autologous bones, thereby reducing removal site morbidity and operating time, and are virtually unrestricted. In connection with the concept of backward planning, they enable aesthetically and functionally successful implant prosthetic results even in the aesthetically sensitive anterior region. Thus, the patient was very satisfied with the surgical procedure and the aesthetic result.

## Data Availability

The data can be requested from the author.

## References

[1] Nilius M., Kohlhase J., Lorenzen J., Lauer G., Schulz M. (2019). Multidisciplinary oral rehabilitation of an adolescent suffering from juvenile Gorlin-Goltz syndrome - a case report. *Head & Face Medicine*.

[2] Chaushu G., Mardinger O., Peleg M., Ghelfan O. (2010). Nissan J. Analysis of complications following augmentation with cancellous block allografts. *Journal of Periodontology*.

[3] Al-Abedalla K., Torres J., Cortes A. R. (2015). Bone augmented with allograft onlays for implant placement could be comparable with native bone. *Journal of Oral and Maxillofacial Surgery*.

[4] Nevins M., Mellonig J. T., Clem D. S., Reiser G. M., Buser D. (1998). Implants in regenerated bone: long-term survival. *The International Journal of Periodontics & Restorative Dentistry*.

[5] Nissan J., Vered M., Gross O., Mardinger O., Chaushu G. (2011). Histomorphometric analysis following augmentation of the posterior mandible using cancellous bone-block allograft. *Journal of Biomedical Materials Research. Part A*.

[6] Schlee M., Dehner J. F., Baukloh K., Happe A., Seitz O., Sader R. (2014). Esthetic outcome of implant-based reconstructions in augmented bone: comparison of autologous and allogeneic bone block grafting with the pink esthetic score (PES). *Head & Face Medicine*.

[7] Nevins M., Mellonig J. T. (1994). The advantages of localized ridge augmentation prior to implant placement: a staged event. *International Journal of Periodontics & Restorative Dentistry*.

[8] Nissan J., Marilena V., Gross O., Mardinger O., Chaushu G. (2011). Histomorphometric analysis following augmentation of the posterior mandible using cancellous bone-block allograft. *Journal of Biomedical Materials Research Part A*.

[9] Krasny K., Krasny K., Kaminski A., Fiedor P. (2015). Global maxillary ridge augmentation with frozen radiation-sterilised bone blocks followed by implant placement: a case report. *Cell and Tissue Ban*.

[10] Rothamel D., Schwarz F., Herten M. (2008). Vertical augmentation of the mandible using cortico-spongious xenoblocks. A histomorphometrical study in dogs. *Schweizer Monatsschrift für Zahnmedizin*.

[11] Schmitt C. M., Doering H., Schmidt T., Lutz R., Neukam F. W., Schlegel K. A. (2013). Histological results after maxillary sinus augmentation with Straumann® BoneCeramic, Bio-Oss®, Puros®, and autologous bone. A randomized controlled clinical trial. *Clinical Oral Implants Research*.

[12] Froum S. J., Wallace S. S., Elian N., Cho S. C., Tarnow D. P. (2006). Comparison of mineralized cancellous bone allograft (Puros) and anorganic bovine bone matrix (Bio-Oss) for sinus augmentation: histomorphometry at 26 to 32 weeks after grafting. *The International Journal of Periodontics & Restorative Dentistry*.

[13] Laino L., Lezzi G., Piattelli A., Lo Muzio L., Cicciu M. (2014). Vertical Ridge augmentation of the atrophic posterior mandible with sandwich technique: bone block from the chin area versus corticocancellous bone block allograft—clinical and histological prospective randomized controlled study. *BioMed Research International*.

[14] Vastardis S., Yukna R. A. (2006). Evaluation of allogeneic bone graft substitute for treatment of periodontal osseous defects: 6-month clinical results. *The Compendium of Continuing Education in Dentistry*.

[15] Tudor C., Srour S., Thorwarth M. (2008). Bone regeneration in osseous defects--application of particulated human and bovine materials. *Oral Surgery, Oral Medicine, Oral Pathology, Oral Radiology, and Endodontics*.

[16] Yacker M., Ricci J., Matei I. C., Hu B., Mamidwar S. (2014). Treatment of a mandibular cyst before implant placement: case report. *The New York State Dental Journal*.

[17] Tolstunov L., Chi J. (2011). Alveolar ridge augmentation: comparison of two socket graft materials in implant cases. *The Compendium of Continuing Education in Dentistry*.

[18] Tallarico M., Park C.-J., Lumbau A. I. (2020). Customized 3D-printed titanium mesh developed to regenerate a complex bone defect in the aesthetic zone: a case report approached with a fully digital workflow. *Materials*.

[19] Nilius M., Mueller C., Nilius M. H., Haim D., Weiland B., Lauer G. (2021). Advanced backward planning with custom-milled individual allogeneic block augmentation for maxillary full-arch osteoplasty and dental implantation: a 3-year-follow-up. *Cell and Tissue Banking*.

[20] Pruss A., Perka C., Degenhardt P. (2002). Clinical efficacy and compatibility of allogeneic avital tissue transplants sterilized with a peracetic acid/ethanol mixture. *Cell and Tissue Banking*.

[21] Hinsenkamp M., Muylle L., Eastlund T., Fehily D., Noël L., Strong D. M. (2012). Adverse reactions and events related to musculoskeletal allografts: reviewed by the World Health Organisation Project NOTIFY. *International Orthopaedics*.

[22] Fretwurst T., Spanou A., Nelson K., Wein M., Steinberg T., Stricker A. (2014). Comparison of four different allogeneic bone grafts for alveolar ridge reconstruction: a preliminary histologic and biochemical analysis. *Oral Surgery, Oral Medicine, Oral Pathology, Oral Radiology*.

[23] Wen S. C., Barootchi S., Huang W. X., Wang H. L. (2019). Time analysis of alveolar ridge preservation using a combination of mineralized bone-plug and dense-polytetrafluoroethylene membrane: a histomorphometric study. *Journal of Periodontology*.

[24] Nissan J., Ghelfan O., Mardinger O., Calderon S., Chaushu G. (2011). Efficacy of cancellous block allograft augmentation prior to implant placement in the posterior atrophic mandible. *Clinical Implant Dentistry and Related Research*.

